# A Review on Toxicodynamics of Depleted Uranium

**DOI:** 10.22037/ijpr.2020.113045.14085

**Published:** 2019

**Authors:** Fatemeh Shaki, Ehsan Zamani, Abdollah Arjmand, Jalal Pourahmad

**Affiliations:** a *Department of Toxicology and Pharmacology, Faculty of Pharmacy, Mazandaran University of Medical Sciences, Sari, Iran. *; b *Department of Pharmacology and Toxicology, School of Pharmacy, Guilan University of Medical Sciences, Rasht, Iran.*; c *Department of Toxicology and Pharmacology, Faculty of Pharmacy, Shahid Beheshti University of Medical Sciences, Tehran, Iran.*

**Keywords:** Depleted uranium, Toxicity, Kinetic, Mechanism, Radiation

## Abstract

Depleted uranium (DU) is an important by product in uranium enrichment process. Due to its applications in civilian and also military activity, DU emerged as environmental pollutant. The exposure to DU can occur via external or internal pathways. In external exposure, mainly beta radiation from the decay products contributes to DU toxicity. Internal exposure to DU is more important and can occur through ingestion of DU-contaminated water and food and inhalation of DU aerosols. There is limited information about health effects and mechanism of DU after environmental exposure. Kidney is reported as the main target organ for the chemical toxicity of this metal that was reported in Persian Gulf syndrome. Alterations in behavior, some neurologic adverse effects, immunotoxicity, embryo-toxicity and hepatotoxicity were observed in chronic exposure to DU. Also, the increased risk of cancer was revealed in epidemiological and experimental studies. Several mechanisms were suggested for DU toxicity such as oxidative stress, mitochondrial toxicity and inflammation. In fact, uranium like other toxic heavy metals can induce oxidative damage and apoptosis via mitochondrial pathway and inflammatory response. In this review, we have discussed the kinetic of DU including source and exposure pathway. In addition, the health effects of DU and also its toxic mechanism have been highlighted.

## Introduction

Uranium is a naturally occurring heavy metal that for the first time was discovered by the German pharmacist Klaroth that isolated uranium from a waste mining ore that known as “pitchblende” (1). Average concentration of Uranium is approximately 3 mg/kg in the earth’s crust that is more common than some metals such as mercury, silver or gold (2).

Uranium then began to be used in the manufacture of ceramic and glass vessels as well as paints. But when Madame Marie Curie isolated radium from uranium and pitchblende, scientific attention turns toward the radiochemistry and radiation physics of this metal. However, health risks of uranium exposure mainly in miners gained attention by physician. In the mid-1500s lung disease was reported in miners work in silver, bismuth and cobalt mining that was already linked to Pitchblende as a waste ore. In fact, the inhalation of uranium dust and radon exposure during mining activity increased the risk of lung cancer in miners (3).

The radio-physical properties of uranium lead to extensive use of this metal in the nuclear weapons field and its use as a fuel in nuclear power reactors. Natural uranium contains mixture of three different isotopes including ^238^U (99.2745%), ^235^U (0.7200%) and ^234^U (0.0055%) (4).

Among three natural uranium isotopes, ^238^U and ^235^U are primordial substances that, via natural decay chains and a series of radioactive daughter products finally converted to a stable lead isotope (Table 1). On the other hand, only ^235^U is a fissile isotope, which can be used both in nuclear power plants and nuclear weapons (5). Uranium with natural isotopic composition used as fuel just in heavy water reactors. But, for other reactor types, this low proportion of fissionable ^235^U (0.7200%) in natural uranium is insufficient. In fact, it is needed to enrich ^235^U levels at least three to five times (or even up to 90%) in natural uranium for using in some reactors (6). 

The enrichment processes separate isotope base on the small differences in masses of particular isotopes. The remaining uranium is referred to as depleted uranium which usually has an isotopic composition of 99.75% ^238^U, 0.25% ^235^U, and 0.005% ^234^U (7).

This change in isotope content also results in differences in the radioactivity of depleted uranium with its natural form. The radio activity of natural uranium is approximately 25.40 mBq/μg and 14.80 mBq/μg for depleted uranium. Thus, the specific activity of depleted uranium as well as its radio toxicity is about 60% of the radioactivity of natural uranium (2, 8). 

Favorable properties of DU such as its very high density of 19.3 g/cm³ (lead: 11.3 g/cm³) making it useful in potential range of applications in the civilian sector such as balance weight in aircraft and in the keel of racing yachts or as shielding material in clinical irradiation facilities. But, high density of DU, combined with its pyrophoric properties, make it an excellent material for the military aims as projectile cores characterized by a very high penetrating power, even for armored targets (9).

DU weapons for the first time were used in the first Persian Gulf War during 1990−1991 and Gulf War Syndrome was reported after the war. Many studies suggested DU exposure as a possible causative agent for this syndrome (10-13). Use of DU munitions continued in the Balkans conflict, the Afghanistan war and the second Persian Gulf War that stimulate scientific efforts for evaluation of DU toxicity and human health risk. So far, radio toxicity of uranium compound was core of attention. But less radioactivity of DU than natural uranium makes focusing on chemical toxicity aspect of DU. In fact, DU has similar chemical behavior with natural uranium and DU can consider as a chemical toxin that may be more of a concern than DU’s radioactivity. The increasing use of this metal in civilian and nuclear industry results in an increasing chronic exposure to low levels of DU by dust inhalation or by dietary intake from contaminated foods or water (14-16).

DU mainly has renal excretion that accumulation of high concentration of DU in kidney tissue during excretion leads to high susceptibility of kidney to DU toxicity (17, 18). The behavior of DU in environment and body and any probable health risks will be discussed in detailed in the following sections.

**DU exposure sources**

The exposure to DU can occur via external or internal pathways. Mainly, beta radiation from the decay products contribute to external exposure of DU. However, the cases of external exposures are very small and the skin is the most affected organ (5). In fact, using of DU in anti-armor weapons is responsible for the main part of external exposure either as DU dust or DU fragments (19). It has been reported that contact dose of DU fragments are about 2 mSv/h and much less is expected for dust. Soldiers in vehicles shielded with DU armor exposed with very low dose rates in the range of 1 µSv/h for longer time periods (2, 5).

Internal exposure to DU is more important and can occur through three pathways including ingestion of DU-contaminated water and food, inhalation of DU aerosols and third pathway that especially is important in soldiers who suffered embedded fragments or contaminated wounds. However, ingestion of DU is not accounted as the main exposure pathway (19). Direct ingestion of DU can occur through hand contamination of contaminated soil or via ingestion of contaminated soil by cattle and sheep and transfer this contamination to humans by consuming animal products (20). Because of small percent of DU-contaminated area, this rout of exposure is negligible (5). On the other hand, intake of drinking water is considered as the major ways of natural uranium exposure (21). Contamination of drinking water with DU can be caused by DU dust, fragments or penetrators buried in soil (22). But, studies on selected sites did not show any contamination of local ground waters, although high amounts of DU were dispersed to the ground (23). Interestingly, the highest concentration of DU was found in suspended sediment carried by run-off water (24). There is general agreement that inhalation of dust is probably the most significant route of exposure both in combat and non-combat situations. 

Exposure to DU aerosols comes from two sources. The first source is due to contact with natural DU in the environment and second source is due to DU munitions used. Once they are fired and impact a target DU munitions form small particulate dust ranging from 0.2−15 microns in diameter (3).

Generally, impact of DU anti-armor weapons on hard targets such as tanks leads to oxidation of DU. The major uranium oxides generated are U_3_O_8_, UO_2_ and UO_3_ (25). These three uranium oxides are relatively insoluble, dissolving only slowly in body fluids (weeks for UO_3_ to years for U_3_O_8_ and UO_2_). After inhalation of these particles, alveolar absorption appears to occur in two phase manner. An early rapid phase which results in peak plasma levels and then a decline followed by a prolonged period of steady absorption. Various particle size and solubility patterns of various oxide forms of DU may be contributed in this biphasic pattern. Also, inflammatory response of the lung tissue can result in retarded absorption after a few days (22). Pulmonary half-life of DU reported about 4 years (26). 

Uranium is absorbed into the blood and then widely distributed throughout the body. DU can be retained by body tissues and organs especially bone. In fact, bone is a reservoir for DU and even after cessation of DU exposure; it will be released from the bone for months or years (17, 18).

**Mechanisms of DU toxicity**

Depleted uranium has ubiquitous property that has both the chemical and radiological toxicities (27). It is suggested that solubility of DU compounds affects its absorption and also toxicity. Soluble chemical forms have more quick absorption than insoluble forms which their absorption generally takes months to years. So, chemical toxicity probably has main role in health effects of soluble forms of uranium while radiotoxicity is more likely is involved in the chronic health effects of insoluble forms which usually can deposit in the lung and local lymph nodes and retained for long time (28).

Radioactivity of DU mainly relates to ^238^U which has very low specific activity and particles of ^238^U release radiation very infrequently. ^238^U decays primarily by alpha emission. Alpha particles have little penetrating power and quickly lose their kinetic energy. If just the alpha emissions take into accounts for radioactivity of DU, only 60% of the radioactivity of natural uranium retained in DU. But, during the decay process of DU, beta and gamma emissions produced by DU daughter isotopes or decay products such as thorium 234 and protactinium -234. It has been shown that beta and gamma particles are more penetrating than alpha emission. But alpha radiation can be dangerous when absorb into body. Deposition of alpha particles in various organs results in emission of its energy in a concentrated area that could leads to greater damage than beta or gamma radiation. In overall, if radioactivity of beta and gamma emissions were considered, the radioactivity of DU calculated as 75% of the natural uranium (7, 29).

While DU has low radioactivity, but it is noteworthy that high amount of DU used during wars. For examples, approximately 300 tons of DU used in the 1^st^ Gulf war and if just a small portion about 1−2% of that were converted to aerosol, three to six million grams of DU dust would be produced. This high volume of dusts would release 1.16 million to 2.32 million Ci of radiation, a measure that would exceed the New York state safety levels for monthly release of 150Ci by a factor of 7,733 to 15,467 (3). Fortunately, the major fraction of DU dust probably sediment in the soil or diluted by the wind. Therefore, much less amount of DU dust would be inhaled or ingested by a population. So, the risk of radioactivity of DU cannot completely ignore or underestimated (3).

On the other hand, the chemical and metallic properties of DU do not differ from natural uranium. Recently, many researchers focused on the chemical aspects of DU toxicity. Although still little is known about the molecular processes leading to DU induced health effects. Between several suggested mechanisms, induction of apoptosis via mitochondrial pathway and inflammatory response is the most important. 

Oxidative stress known as an imbalance between free radical generation and the antioxidant defense system in cells (30). Oxidative damage in cells is considered a common threat and danger for all aerobic organisms. ROS (reactive oxygen species) can be generated by endogenous physiological mechanisms or after exposure to exogenous compounds such as environmental toxin (31, 32).

Several studies revealed that uranium like other heavy metals can be considered as a mitochondrial toxin and can induce mitochondrial dysfunction in different target organs (33-36).

It is well known that mitochondria are the main source of ROS generation and are also the major cellular site of energy production in the cells. Therefore, normal function of mitochondria is essential to maintain brain homeostasis (36).

Shaki *et al.* showed that DU is capable to disrupt electron transfer chain via inhibition of complex I and III that leads to increase in ROS generation in cells (10). DU-induced oxidative damage to mitochondria leads to the loss of mitochondrial membrane potential in a dose-dependent manner and finally results in initiation of apoptosis signaling (37, 38). Moreover, another studies showed that DU exposure leads to activation of caspase-3, caspase-8 and caspase-9. Therefore, both a mitochondria-dependent signaling pathway and a death receptor pathway by a crosstalk have role in DU-induced apoptosis (33).

Another suggested molecular mechanism for concerning the pathological effects of DU is involvement of inflammatory processes. In fact, inhalation and deposition of particulate matter leads to exposure of these particles with macrophages as the main target cells in inflammatory process (39). Macrophages are involved in particle clearance and retention in the alveolar compartment (40). Activated macrophages are known to secrete different mediators: pro- and anti-inflammatory cytokines. Although the inflammatory response has the key role in the host defense system but excessive or persistent inflammation contributes to the pathogenesis of disease (41). 

Several studies demonstrated the increase of inflammatory cytokine expression after *in-vivo* or *in-vitro* exposure to DU (42). *In-vitro* studies with DU exposure on macrophages have shown an induction of TNF-a (tumor necrosis factor- alpha) secretion and MAPK (mitogen- activated protein kinase) activation (43, 44). Activation of cytokine expression and/or production was noted either in pulmonary tissues following uranium exposure by inhalation (42). Also, simulation of prostaglandins was also reported in the kidney after acute contamination (45, 46).

Another study demonstrated that chronic ingestion of DU induced time-dependent modifications of inflammatory pathways, notably in terms of immune cell content. The ultimate effects of DU contamination might be pathogenic by suppressing defense mechanisms or inducing hypersensitivity (47). One very recent study reported that toxicity of U (VI) is concentration dependent on human lymphocytes which are very important cells in conducting immune response. Based on their report, U (VI) induced cell death and oxidative stress via increased ROS production, mitochondrial and lysosomal membrane destabilization, glutathione depletion and lipid peroxidation in human lymphocytes. DU-induced mitochondrial damages was associated with activation of apoptosis in lymphocytes which concludes severe suppression of immune system in mammalians following exposure to environmental concentrations of DU (48).

**DU Toxicity: health effects**

**Figure 1:**
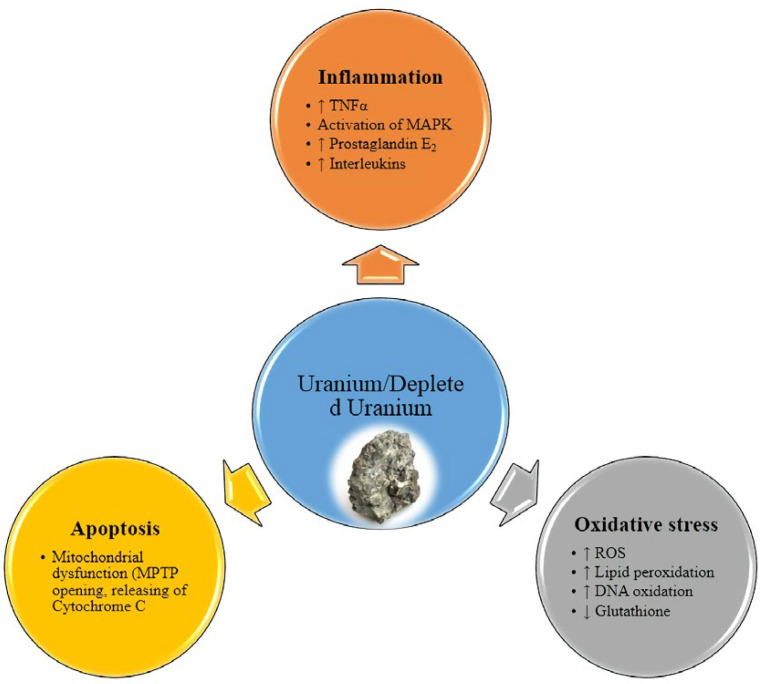
Mechanism of uranium and depleted uranium toxicity

After using of DU ammunition on battle fields about 10 years ago, DU has been repeatedly suggested as the cause of cancer, leukemia and other health effects among people who were present at site where DU ammunition had been used during conflicts such as Gulf War, Kosovo, Bosnia and Montenegro civil conflicts. The most important health effects on human exposure or revealed toxicity in experimental studies discussed in following sections (Figure 1).

*DU and Cancer*

Animal studies showed evidence of carcinogenesis of uranium compounds for animals (49, 50). For example, in one study inhalation of uranium oxide caused lung cancer in monkey after long term exposure. The mechanism of DU carcinogenicity is not clear, but probably DU can induce DNA double strand breaks in rats (42). Studies showed that small DU foils embedded in muscle tissue could elevate risk of cancer in the rats (50).

DU exposure caused soft tissue sarcomas in rats and induction of genetic changes in mouse macrophages (51, 52). Also, in Ames test, DU increased urinary mutagenicity (53). Increased DNA methylation was reported in a rat model of DU-induced leukemia (54).

*In-vivo* studies with embedded DU pellets in animals showed aberrant expression of oncogenes and tumor suppressor genes associated with carcinogenesis (55).

Also, it is found that after exposure of mice to embedded DU for 3 months then injected with progenitor cells, 75% of mice developed leukemia (compared with 10% in control mice). In addition, mice showed changes in the musculoskeletal system, *i.e.* bone formation and remodeling, after oral, intraperitoneal, intravenous and implantation uranium exposure (26).

*DU nephrotoxicity*

Uranium is a well-established nephrotoxin (*i.e.* it is toxic to kidneys) in humans, the primary target being the proximal tubule. The kidneys are the critical organ for uranium chemical toxicity. For high acute exposures, precipitation of uranyl-carbonate complexes in the proximal tubules may lead to impairment of kidney function and irreversible damage at very high exposures (27). Damage occurs when uranium forms complexes with the phosphate ligands and proteins in tubular walls, which impair kidney function. Biomarkers of these tubular effects include enzymuria and increased excretion of small proteins, amino acids and glucose. Chelating compounds may be used to prevent or reduce kidney damage in such accidental situations (25). In US soldiers with a high load of DU shrapnel, no indications of kidney dysfunction were seen in tests made several years after the Gulf War. 

*Neurological effects of DU*

Much of our knowledge of the human effects of DU comes from studies examining Gulf War veterans exposed to DU that often have problems such as small sample size and diverse nature of soldiers. Therefore, because of limited data about human exposure to DU, there are not any long term studies addressing neuropsychological effects of DU exposure. In animal studies, exposure of adult animals to DU lead to subtle but important changes in behavior, including increased activity in a test apparatus and impaired working memory. These behavioral changes correlate with DU induced lipid peroxidation seen in the brain (10, 56).

To date, all our knowledge about the potential neurodevelopmental effects of uranium comes from experimental work on animals. Administration of low doses of uranium (insufficient for making renal damage) to pregnant animals produced small litters, smaller offspring size, increased offspring mortality and skeletal abnormalities (7). 

Exposure of developing animals to DU in drinking water which simulate long term human dosage and exposure to DU leads to subtle derangements of behavior. In rodents, exposure to DU during development actually accelerates the appearance of a number of behaviors (righting reflex, forelimb placing, grasping, swimming and weight gain) (57).

Administration of DU to adult animals during development stage, showed low performance at working memory test and induced smaller brain weights (as a percentage of body weight). In general, there is not sufficient data about effects of DU on neurodevelopmental toxicity in humans or any other primate species (3).

*Embryo toxicity of DU*

Based on the latest literature there are only a few articles which have studied the embryo toxicity effects of this metal on mammals. In one of these studies, doses of 0, 5, 10, 25 and 50 mg/kg/day of Uranium acetate dehydrate was taken to pregnant mice during the pregnancy period of 15-6 days. Toxic effects reported as weight loss, reduced food intake and growth retardation in pregnant mice that did not depend on the effects of uranium toxic doses, but doses by more than 5 mg/kg/day were caused toxicity in embryos. Cleft palate, hematoma, loss of bone in the skull of the fetus were the effects of uranium toxicity which is reported in this study (58). In another study, maternal toxicity, reduced weight and increased relative liver weight was observed. Fetal toxicity consisted of decreased fetal body weight, body length, cleft palate, and mass region of blood on face was seen. Problems such as rotation and reduced ossification of skull and bones, tail, hind foot and paragraphs Metatarsus the front toes were exist (59).

In another research work the Depleted Uranium with different concentrations was implanted in the body of pregnant rats, the female mice were mated and pregnant, at twentieth day of pregnancy the embryo were taken outside the body of all pregnant mice the level of Uranium in liver, kidney and fetal plasma were measured. Although uranium levels were increased in fetal body, but no specific toxicity such as weight loss, and small size were observed (60).

As “Bertel” mentioned in Teratogenic Toxicity: “Soluble Uranium oxide and Nano-particles can cross the placenta to the fetus and rapidly toxic the growing fetus. At low doses, Nano-particles damage the fetal brain and cause mental retardation and behavioral problems. Other teratogenic effects include various deformities and diseases, and even affect the growth hormone and the immune system of fetal. In the research was carried out male rats, were received Uranium acetate dehydrate with doses (0, 5, 10 and 25 mg/kg day) by gavages for 60 days then mated with females that had been treated for 14 days. Growth and development of infants were studied after 0, 4 and 21 days of lactation; in this period the increases in the number of dead infants were observed (61).

The potential adverse effects of DU in pregnant women who were exposed to depleted uranium. in the recent 60 years was including; 1- Female victims of using banned bombing weapons containing depleted uranium in countries such as Iraq, Kuwait, Afghanistan. 2- Women employed in civilian nuclear reactors, uranium mines and mining. 3- Women who participate in the process of nuclear waste or living near a nuclear waste disposal site, was always one of the most serious concerns’ in environmental toxicology.

And finally in a recent study carried out by our team (62) the histological results showed: 

Lack of specific curve in cerebral cortex.

In one case the fetus did not have fingers in hand.

The rotation of C shape in fetus of test group did not observe and their backs were straight.

The weight of fetuses and the size C-R were more than control group.

The appearance of liver was large and dark.

Disappearing of umbilical hernia in 15 days’ fetus is the reason of delay in growth of it.

In our study fetuses were weighted at 15 day of gestation (62). The weight and the length of fetuses in test group were significantly higher than those of Sham and control groups which is quite in appose to the Domingo findings in 1989 (59), which reported significant decrease in weight of fetuses whose mothers were exposed to depleted Uranium. 

Having considered other parts of results that the liver of fetuses in DU treated group were larger and head of them also were larger than those of control and Sham groups, we can conclude that our findings seem more logical than Domingo findings in 1989 (59).

*Hepatotoxicity of DU*

Liver is considered as another target of DU in human. Previous experimental studies exhibited accumulation of DU in the liver tissue (63). Various works reported that DU via modulation of cytochrome P450 (CYP) activity can affect the metabolism of bile acids as well as xenobiotic in liver (64). 

Indeed, some changes in histological and biochemical markers have been reported in DU-exposed mice. Yapar *et al.* showed a significant liver damage which was associated with the increased levels of alanine aminotransferase, aspartate aminotransferase and pathological changes in liver tissue of mice after treatment by DU (65).

Pourahmad *et al.* showed that exposure of rat hepatocytes with DU lead to significant increase of ROS generation, glutathione oxidation, lipid peroxidation, mitochondrial membrane potential collapse, and lysosomal membrane rupture. Also, they suggested that impairment in mitochondrial/lysosomal function lead to DU-induced cytotoxicity in rat hepatocyte (37, 66).

In another study, Shaki *et al.,* demonstrated that DU inhibited mitochondrial complex II activity and result in succinate-supported mitochondrial ROS production and elevation of oxidative stress markers in liver isolated mitochondria. Therefore, DU-induced hepatic toxicity might be mediated via mitochondrial oxidative damage and uncoupling of oxidative phosphorylation (36, 38).

**Table 1:** Comparison of Natural and depleted uranium isotops

**Nuclide**	**Half-life (years)**	**Natural Uranium**		**Depleted Uranium**	
		**weigh percent**	**Activity** **[kBq/g]**	**weigh percent**	**Activity** **[kBq/g]**
^238^U	4.47 × 10^9^	99.27	12.4	99.75	12.4
^235^ U	7.04 × 10^8^	0.72	12.4	0.25	2.26
^234^ U	2.45 × 10^5^	0.005	0.6	0.005	0.16
Total activity		25.4		14.8	

## Conclusion

Depleted uranium is a by-product of uranium enrichment that mostly known as a chemical toxic element. Military use of DU were important way of environmental exposure. The two main roots of exposure to DU are ingestion of contaminated drinking water and inhalation of polluted dust (especially in combat situation). This study has discussed some toxicological aspects of DU. Nephrotoxicity of uranium is its well-known toxicity. But, carcinogenicity, neurotoxicity, embryo toxicity and hepatotoxicity are other reported adverse effects due to DU exposure. Furthermore, it was suggested that DU like other toxic heavy metals can induce oxidative stress and mitochondrial dysfunction. In fact, the most important mechanism which is involved in DU-induced toxicity is triggering apoptosis via mitochondrial pathway and inflammatory response. Nevertheless, Other health effects of uranium need to be better studied.
